# Adaptation of Interspecific Mesoamerican Common Bean Lines to Acid Soils and High Temperature in the Amazon Region of Colombia

**DOI:** 10.3390/plants10112412

**Published:** 2021-11-09

**Authors:** Juan Carlos Suárez, Milan O. Urban, Amara Tatiana Contreras, Miguel Ángel Grajales, Cesar Cajiao, Stephen E. Beebe, Idupulapati M. Rao

**Affiliations:** 1 Programa de Ingeniería Agroecológica, Facultad de Ingeniería, Universidad de la Amazonia, Florencia 180002, Colombia; amaratatis18@gmail.com; 2 Programa de Maestría en Sistemas Sostenibles de Producción, Facultad de Ingeniería, Universidad de la Amazonia, Florencia 180002, Colombia; 3 Centro de Investigaciones Amazónicas CIMAZ Macagual César Augusto Estrada González, Grupo de Investi-gaciones Agroecosistemas y Conservación en Bosques Amazónicos-GAIA, Florencia 180002, Colombia; 4 International Center for Tropical Agriculture (CIAT), Km 17 Recta Cali-Palmira, Cali 763537, Colombia; m.urban@cgiar.org (M.O.U.); m.a.grajales@cgiar.org (M.Á.G.); c.cajiao@cgiar.org (C.C.); s.beebe@cgiar.org (S.E.B.); i.rao@cgiar.org (I.M.R.)

**Keywords:** energy use, leaf cooling, phenology, photosynthesis, photosynthate partitioning, pollen viability

## Abstract

Knowledge of the physiological basis for improved genetic adaptation of common bean (*Phaseolus vulgaris* L.) lines to acid soils and high temperature conditions in the Amazon region of Colombia is limited. In this study, we evaluated the differences among 41 common bean lines in energy use, leaf cooling, photosynthate partitioning to pod formation and grain filling, and grain yield over two seasons under acid soil and high temperature stress in the Amazon region of Colombia. Common bean lines evaluated included medium and large seeded interspecific lines of Mesoamerican and Andean gene pools with different levels of adaptation to abiotic stress conditions and some lines are improved for iron and zinc (biofortified) concentration in seeds. We found three bean lines (GGR 147, SMG 21 and SMG 12) that were superior in their photosynthetic response, leaf cooling, photosynthate partitioning ability to pod formation and grain filling, resulting in grain yields exceeding 1900 kg ha^−1^ under acid soil and high temperature stress conditions. The superior photosynthetic performance was attributed to the efficient use of absorbed energy on the electron level in thylakoids, which is mainly oriented to a higher quantum yield of PSII (ΦII), lower energy dissipation in the form of heat (ΦNPQ), high linear electron flow (LEF) and high fraction of PSI centers in open state (PSIopen). We speculate that these photosynthetic and photosynthate partitioning responses of superior bean lines are part of the genetic adaptation to acidic soils and high temperature stress conditions. Among the evaluated bean lines, three lines (GGR 147, SMG 21 and SMG 12) combined the desirable attributes for genetic improvement of stress tolerance and biofortification. These lines can serve as parents to further improve traits (energy use efficiency and multiple stress resistance) that are important for bean production in the Amazon region.

## 1. Introduction

The common bean (*Phaseolus vulgaris* L.) is one of the five cultivated species of the genus *Phaseolus*, and it is ranked as one of the main grain legumes after soybean and peanut [1]. It plays an important role in improving food security and income generation [2,3]. Beans are high in protein, fiber, and carbohydrates, as well as vitamins and minerals [1,3,4,5]. The higher mineral concentration (Ca, Mg, K, Cu, Fe, Mg and Zn) in seed is one of the main characteristics of bean domestication in different regions of Central America, the Andean region of South America and the eastern and southern regions of Africa [6,7,8].

The world’s bean-producing land is affected by edaphic stress factors such as low soil fertility [9] or soil acidity and aluminum (Al) toxicity [10]. These areas are also affected by climatic stress factors caused by seasonal drought and high temperatures or excess rainfall [10,11,12,13,14], resulting in yield losses reaching 80–100% [5,13,15]. Bean breeding programs have been making progress in identifying the adaptive responses that contribute to an increase in yield under stress conditions [12,16,17,18,19]. In addition to developing advanced lines capable of adapting to acid soils [9] and high temperatures [11], it is necessary to evaluate new lines for their capacity in increasing efficiency in energy use, leaf cooling and photosynthate partitioning for pod formation and grain filling as well as mineral accumulation in the seed [10,20].

Beans have been classified into two major gene pools based on seed size differences, with large-seeded beans being Andean (South America) and small-seeded beans being Mesoamerican (Central America) in their origin [21,22,23], gene pools with a large number of very diverse accessions spatially distributed from western to eastern parts of southern Europe [24]. These stocks are widely distributed in other regions of the world, such as Europe, whose characterization results show the domestication pathways of the common bean and the runner (*Phaseolus coccineus* L.) [25,26]. These two gene pools (Andean and Mesoamerican) differ in many aspects, including some physiological responses as well as nutritional characteristics [27]. Andean beans tend to have a higher seed iron concentration than the Mesoamerican beans [2,22] but their level of tolerance to abiotic stress tends to be lower [18]. Improved bean genotypes with higher levels of iron (Fe) and zinc (Zn) have been released in recent years in different regions of the world [20,28]. These new bean varieties are not only biofortified for higher iron and zinc concentration but also perform agronomically as well as standard varieties due to a combination of multiple desirable traits including good yield, color, size and shape of the grain; characteristics that are desired by farmers [20,29]. 

In the Amazon region, previous research resulted in the identification of common bean genotypes that are adapted to low fertility acid soils [9] and high temperature stress [11]. These studies included small seeded Mesoamerican and large seeded Andean bean lines that were developed for improving tolerance to acid soils, drought and heat stress. We have used different phenotypic variables related to biomass production, dry matter partitioning indices, grain yield, yield components [15] as well as traits related to plant efficiency [30] that focus mainly on energy use in leaves (i.e., energy that passes to photochemistry ΦII, dissipates as heat ΦNPQ and dissipates in an unregulated manner ΦNO) and leaf cooling. Using this approach, we have been able to identify several bean lines that are adapted to Amazonian acid soil and heat stress conditions. The better adapted lines showed not only efficient photoassimilate mobilization capacity for pod formation and grain filling, but also combined leaf cooling and greater energy use and pollen viability as adaptive responses to heat stress [31,32]. Energy use is related to the functioning of the photosynthetic apparatus which can be evaluated through measurements on linear electron flow (LEF), conductance (gH+) and proton flux of the thylakoid membrane (νH+), proton motive force (pmf) estimated from transthylacoid membrane proton transport (ESCt) [33], and the different redox states of PSI [34]. A MultispeQ device [35] linked to the PhotosynQ platform (http://www.photosynq.org (accessed on 26 October 2021)) has been used to make a large number of these non-invasive measurements in a short period of time and this tool has been widely used for phenotyping in different crops [31,32,36,37,38]. 

The main objective of the present study was to evaluate differences among the medium and large seeded Mesoamerican and Andean common bean lines in energy use, leaf cooling, photosynthate partitioning to pod formation and grain filling, and grain yield over two seasons under acid soil and high temperature stress in the Amazon region of Colombia. We tested the hypothesis that the well-adapted common bean lines will combine greater energy use and leaf cooling with greater partitioning of photosynthates to pod formation and grain filling under acid soil and high temperature stress. 

## 2. Results

### 2.1. Genotypic Differences in Agronomic Performance

**Figure 1:**
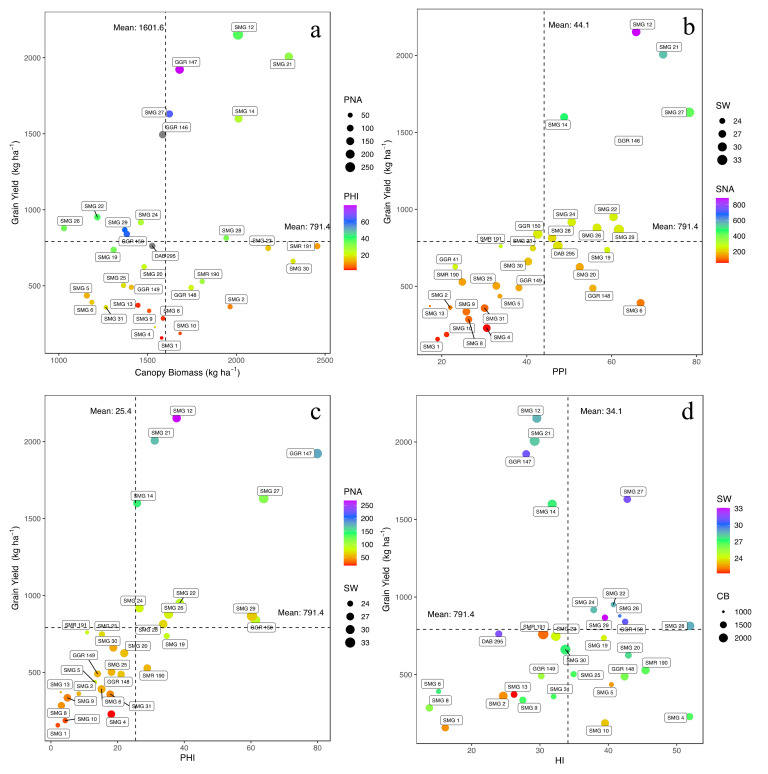
Relationship between grain yield (GY) and different biomass partitioning indices as a function of gradient (change in the color of violet and red from higher to lower value) and magnitude (size of the circle), of parameters that affect the response variable. The dotted lines on the axes correspond to the means of the dependent and independent variables. (**a**) Grain Yield (GY) and Canopy Biomass (CB) as a function of Pod Harvest Index (PHI) and Pod Number Area (PNA). (**b**) Grain Yield (GY) and Pod Partitioning Index (PPI) as a function of Seed Number Area (SNA) and Seed Weight (SW). (**c**) Grain Yield (GY) and Pod Harvest Index (PHI) as a function of Pod Number Area (PNA) and Seed Weight SW. (**d**) Grain Yield (GY) and Harvest Index (HI) as a function of Seed Weight (SW) and Canopy Biomass (CB).

Grain yield (GY) of the 41 evaluated genotypes ranged from 157 to 2152 kg ha^−1^ with a mean value of 791 ± 93 kg ha^−1^. Lines GGR 147, SMG 21 and SMG 12 had higher yields, exceeding 1900 kg ha^−1^ (Supplementary Material Table S1). However, lines such as SMG 1, SMG 10, SMG 4 and SMG 8 presented the lower yields that were below 300 kg ha^−1^ (*p* < 0.001, Supplementary Material Table S1). We found that canopy biomass (CB) values ranged from 994.9 to 2455.9 kg ha^−1^ with an average value of 1602 kg ha^−1^. The lines with the highest CB values were SMR 191, SMG 30, SMG 21, SMG 23, SMG 14, and SMG 12 (*p* < 0.001, Supplementary Material Table S1). Similarly, the lines that presented higher values for both CB and GY were SMG 12, SMG 21, GGR 147, and SMG 14 and these lines also presented higher values of pod partitioning index (PPI) (more than 50%) as well as number of pods per area (PNA) (more than 200 pods m^−2^) (*p* < 0.001, Figure 1a). Other genotypes such as SAP 1, SMG 26, SMG 5, SMG 6, and SMG 11 showed lower capacity to produce CB and also lower efficiency in the remobilization of photoassimilates as revealed by the dry mater partitioning indices of pod partitioning index and pod harvest index (PHI) (Supplementary Material Table S1). The PNA values ranged from 19.18 to 268.1 pods m^−2^, and four genotypes (SMG 12, SMG 21, GGR 147 and SMG 12) presented higher number of pods per area (Figure 1a, Supplementary Material Table S1). Among the four genotypes, SMG 12 was outstanding with more than 200 pods m^−2^.

In relation to the mobilization of photoassimilates from vegetative structures to pod formation, the PPI values ranged from 17.2 to 78.3% (Figure 1b, Supplementary Material Table S1), with SMG 27, SMG 21, SMG 6, SMG 12, DAA 129, GGR 146, SMG 29, and SMG 22 being the bean lines with PPI values above 60% (*p* < 0.001). For the mobilization of pod wall assimilates toward grain formation and grain filling as reflected by PHI values, lines such as SMG 29, GGR 150, SMG 27 and GGR 147 stood out with values of more than 60% (Figure 1c, Supplementary Material Table S1). The harvest index (HI) values ranged from 13.8% to 51.9% (Figure 1d, Supplementary Material Table S1), where only two lines (SMG 4 and SMG 28) transformed more than 50% of the biomass of vegetative structures into grain formation (*p* < 0.001). For the seed number per area (SNA), the genotypes SMG 21, GGR 47 and SMG 12 stood out for producing more than 500 seeds m^−2^ (*p* < 0.001). Lines such as SMG 4, SMG 1, SMG 10 and SMG 31 presented values lower than 100 seeds m^−2^. The average weight of 100 seeds was between 21.3 and 33.1 g, with lines GGR 150, GGR 147, SMG 27, DAB 295 and SMG 29 showing values greater than 30 g (*p* < 0.001).

### 2.2. Genotypic Differences in Phenology and Viability of Pollen under High Temperature Stress

Days to flowering, in the two seasons in which the adaptation of different bean lines to acid soils and high temperature stress was evaluated, ranged from 29 to 34 days after planting (DAP). Lines such as SMR 190, GGR 149 and SMG 28 were earlier to flower as a stress response (Supplementary Material Table S1). Lines such as SMG 23, GGR 150, SMG 24, SMG 21 were earlier to reach physiological maturity. However, an inverse relationship was found between GY and DPM. SMG 21, GGR 147 and SMG 12 were the three genotypes with higher values of GY but these took longer time to reach the growth stage of physiological maturity (*p* < 0.001, Supplementary Material Table S2). Pollen viability values ranged from 35.9% to 82.2% with a mean of 55.7%. Genotypes such as SMG 11, SMG 6, GGR 149, SMG 5, SMG 4, SMG 25, SMG 2, SMG 31, SMG 10 and SMG 8 showed pollen viability values that were greater than 70% (*p* < 0.001, Supplementary Material Table S1). However, lines such as DAB 295, DAA 129 and SMR 168 had pollen viability values that were lower than 40% (Supplementary Material Table S1).

### 2.3. Genotypic Differences in Energy Use and Leaf Cooling under High Temperature Stress

**Figure 2:**
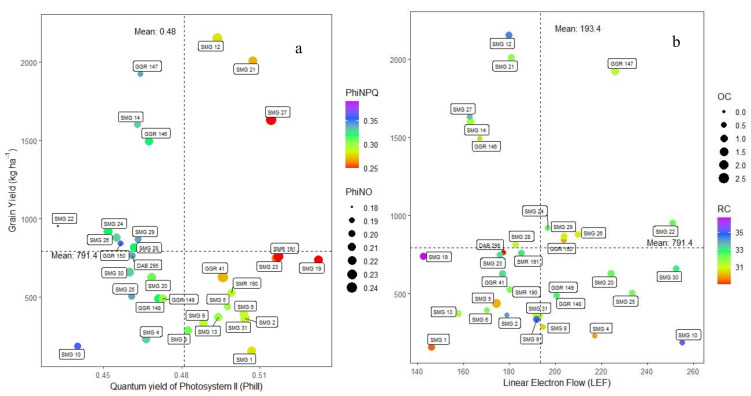
Relationship between grain yield (GY) and different variables related to the functioning of the photosynthetic apparatus. (**a**) Grain yield and quantum yield of photosystem II (ΦII) as a function of the gradient of non-photochemical quenching ΦNPQ (change in the colour of violet and red as greater to lesser value) and magnitude of non-regulated processes ΦNO (size of the circle), parameters that affect the response variable. (**b**) Grain yield and linear electron flow (LEF) as a function of the gradient of PSI Over Reduce Center PSIor (change in the colour of violet and red as greater to lesser value) and magnitude of PSI Open Center PSIopen (size of the circle), parameters that affect the response variable. The dotted lines on the axes correspond to the means of the dependent and independent variables.

When analyzing the differences in photosynthetic apparatus performance it was found that parameters such as LTD, LEF, ECSt, vH+ and PSIact were significantly different between lines (*p* < 0.001). In general, all lines evaluated were able to adjust leaf temperature below the ambient value. For example, lines such as SMG 27 and SMG 23 presented more negative LTD values indicating marked leaf cooling under high temperature stress. However, other lines such as GGR 148 presented less negative LTD values and their energy use efficiency related to photosynthetic processes was also low. For LEF parameter, line SMR 168 presented the highest value while SMG 10, SMG 22 and SMR 168 presented higher values for both ECSt and vH+ parameters. Line SMR 190 showed the highest value for PSIact (Supplementary Material Table S2). Parameters derived from chlorophyll fluorescence were significantly influenced by acid soil and high temperature stress (*p* < 0.001). For example, higher values of GY were related to higher values of PSII quantum yield (ΦII), a situation presented with two lines, SMG 12 and SMG 21, and these lines also exhibited lower value of ΦNPQ (Figure 2a). However, stress effects were obviously showed in most of the bean lines due to the increase in ΦNPQ and a lower distribution of energy to photochemical processes (ΦII) that negatively impacted grain yield and theses lines yielded below 1000 kg ha^−1^ (*p* < 0.001, Figure 2a). When analyzing the relationship between GY and LEF, line GGR 147 (GY= 1900 kg ha^−1^) showed a higher electron transport rate combined with a higher proportion of open PSI centers (LEF = 226.2) (Figure 2b). The opposite relationship was observed with lines SMG 12 and SMG 21, which showed yields of above 2000 kg ha^−1^ with lower LEF values (179.8 and 180.9, respectively) and higher ΦNO values (Figure 2a,b, Supplementary Material Table S2). In addition to the presented trend between GY and LEF, lines SMG 12 and SMG 21 had higher PSI Open Center (PSIopen) and PSI Over Reduce Center (PSIor) values (Figure 2b). 

**Figure 3:**
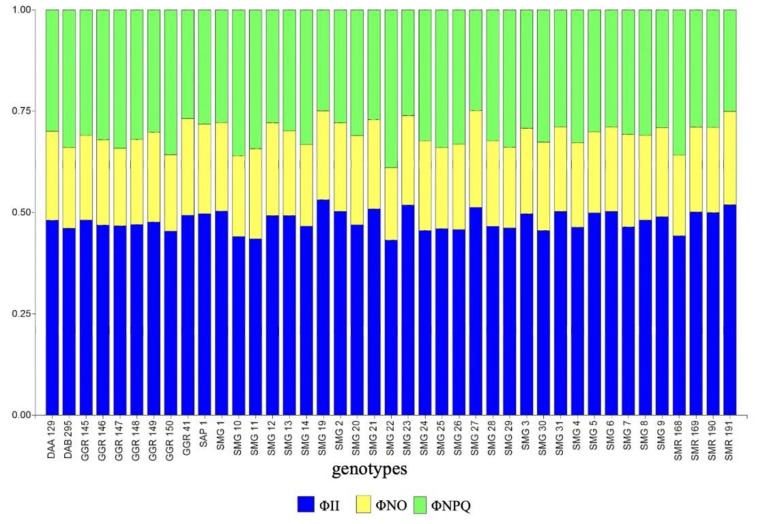
Genotypic differences in energy allocation proportions. Each bar represents the unit, and it is split into three parts in terms of the complementary quantum yields of PSII: ΦII: quantum yield of photochemical energy conversion in PSII, ΦNO: quantum yield of non-regulated non-photochemical energy loss in PSII, and ΦNPQ: quantum yield of regulated non-photochemical energy loss in PSII (ΦNPQ).

**Figure 4:**
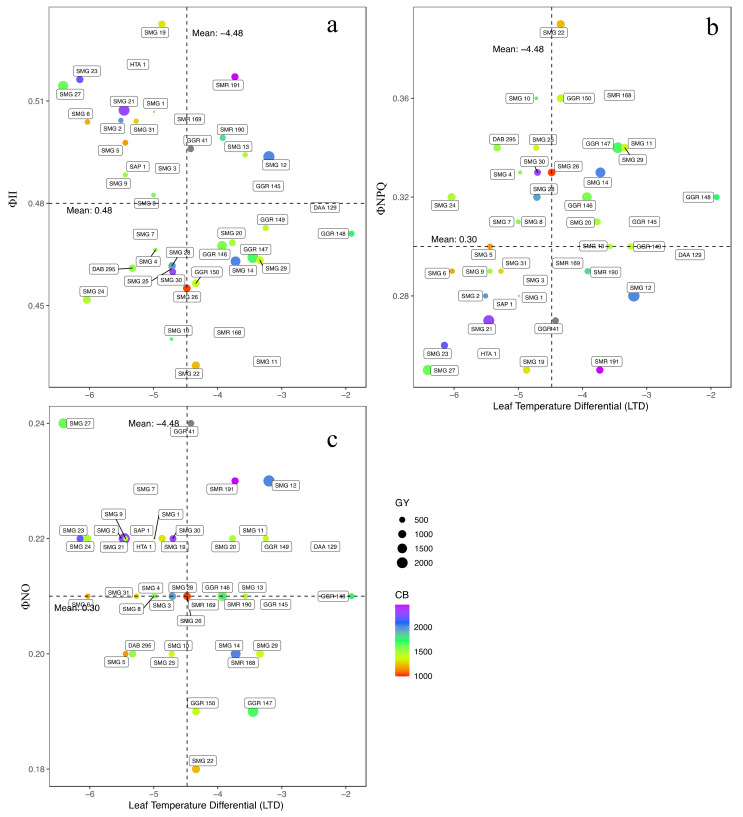
Relationship between different energy pathways and Leaf Temperature Differential (LTD) as a function of the gradient of non-photochemical quenching CB (change in the colour of violet and red as greater to lesser value) and magnitude of non-regulated processes GY (size of the circle), parameters that affect the response variable. (**a**) Quantum yield of photosystem II (ΦII) and LTD; (**b**) Quantum yield of regulated non-photochemical energy loss in PSII (ΦNPQ) and LTD; (**c**) Quantum yield of regulated non-photochemical energy loss in PSII (ΦNPQ) and LTD. The dotted lines on the axes correspond to the means of the dependent and independent variables.

In terms of energy use, the bean lines, on average, used 48.1% and 30.7% for photosynthetic processes (ΦII) and dissipation of energy in the form of heat (ΦNPQ) and the remaining in unregulated processes (21.2% ΦNO) (Figure 3). When we analyzed the chronic effect of temperature on the photosynthetic apparatus, we found very particular behavior such as those presented for SMG 12, SMG 21 and SMG 27. These three lines presented a level of ΦII above 49.4%, which positively impacted agronomic performance variables such as CB and GY, and these differences were closely associated with leaf cooling by showing the values of −3, −5 and −6 °C of LTD for SMG 12, SMG 21 and SMG 27, respectively (Figure 4a). However, lines such as SMG 1, SMG 23, SMG 19, SMG 2, SMG 31 and SMG 5 presented mechanisms in the use of energy as a way to eliminate the negative effect of high temperature on thylakoid membranes. However, these lines did not present an efficient capacity to mobilize photoassimilates for grain formation and grain filling as it was observed with SMG 12, SMG 21, and SMG 27 (Figure 4a). When comparing in detail the behavior of the photosynthetic apparatus of the bean lines that presented the highest grain yield, differences that are mainly related to the ability to dissipate heat were found. For example, the SMG 12 line presented an LTD value of 3°C higher (less negative) than the general average of SMG 27 genotype, but with significantly higher grain yield due to its capacity to translocate photoassimilates. This is probably due to the higher efficiency of PSII as reflected by the ability to regulate excess energy by increasing the efficiency of the electron transport chain (ΦII Figure 4a), thus dissipating energy in a lower proportion in the form of heat (ΦNPQ Figure 4b). However, SMG 21 and GGR 147 presented lower values of ΦNO while GGR 147 showed a higher LTD value (Figure 4c).

### 2.4. Correlations between Different Physiological and Agronomical Performance Traits

According to the correlation analysis it was found that pollen viability (PV) was negatively related to different biomass partitioning indices (PPI, PNA, SNA) and to GY, as well as LTD (Table 1). For example, GY was correlated with CB (r = 0.33 *p* < 0.05), PPI (r = 0.74, *p* < 0.05), PHI (r = 0.68, *p* < 0.05), PNA (r = 0.92, *p* < 0.05), and SNA (r = 0.96, *p* < 0.05) (Table 1). Similar trend was found for PHI with the variables PPI (r = 0.68, *p* < 0.05), HI (r = 0.38, *p* < 0.05), PNA (r = 0.55, *p* < 0.05) and SNA (r = 0.59, *p* < 0.05) (Figure S1 in the Supplementary Material). In general, bean lines that presented higher CB values also presented higher SNA values. The value of RH (relative humidity) correlated positively with DMP (Table 2) while ET (environmental temperature) was negatively correlated with PNA and SNA. LTD was positively correlated with SNA and DMP (Table 2).

When analyzing the relationship between the partitioning indices and the functioning of the photosynthetic apparatus, we found very important results in relation to the adaptability of the different bean lines under conditions of acid soils and high temperatures. For example, leaf cooling (LTD) influenced the number of grains (SNA, r = 0.28) as well as phenological adjustment (DPM, r = 0.38) and pollen viability (PV, r = −0.3) (Table 2). At the level of the functioning of the photosynthetic apparatus, we found that temperature had a negative effect on the proton conductivity of the thylakoid membrane (gH + , r = −0.3) as well as on the energy that is directed to photosynthetic processes (ΦII, r = −0.34, Table 2). This correlation is supported by the low values found for gH+ and ΦII in one of our control lines (DAB 295, Table 2). Likewise, in response to chronic heat stress, as an adaptation strategy the bean lines dissipated energy in the form of heat (ΦNPQ, r = 0.31; NPQt, r = 0.34) as well as the transport of protons in the thylakoid membrane (ECSt, r = 0.31) (Table 2. Adaptation in the functioning of the photosynthetic apparatus (vH+, ECSt, LEF, ΦII) translated into positive relationships with biomass partitioning indices (HI, PHI) in some genotypes; however, the redox states of the PSI affected both positively (PSIor, r = −0.38) and negatively (PSIox, r = −0.38) the HI (Table 2).

## 3. Discussion

### 3.1. Remobilization of Photoassimilates Contributes to Improved Agronomic Performance of Interspecific Lines under Acid Soil and High Temperature Stress

In crops such as the common bean, where grain is the product of interest, the main selection criteria are focused on agronomic performance, which is reflected through genetic improvement in grain yield [5]. This study provides data on physiological evaluation of several advanced lines of common beans developed through interspecific crosses between different species of the genus *Phaseolus* for improving stress tolerance and seed size and marketable color. Previous research by Suárez et al. [9,11] indicated that shoot traits such as CB and dry matter partitioning indices (PPI, PHI and HI) are useful to evaluate genotypic differences in adaptation to acid soils and high temperature stress in the Amazon region. 

High-yielding lines such as SMG 12 and SMG 21 showed higher values of CB compared to other lines. This may be due to their greater ability in net CO_2_ fixation capacity, nutrient assimilation, and water use under heat stress conditions [14]. Other lines such as SMG 14, SMG 23, SMG 30, and SMR 191 also showed higher CB values (>2000 kg ha^−1^) but their grain yield values were not that high which is likely due to increased sensitivity to high temperatures that impacted pod formation and grain development. Only two lines, SMG 4 and SMG 28 showed higher values of HI (>0.5) and among these two lines, the latter was early maturing with GY value higher (813 kg ha^−1^) than the average (791 kg ha^−1^).

An important trait for improving the adaptation of common bean lines under stress conditions is the ability to redistribute stored assimilates to grain [39,40]. Several studies indicated that superior performance under abiotic stress conditions is associated with more efficient photoassimilate mobilization to pods and enhanced grain formation [5,9,11,12,15,19,41,42]. In this regard, lines such as SMG 12, SMG 21, SMG 22, SMG 27, and SMG 29 showed higher values of PPI (higher pod biomass to canopy biomass ratio) but among these lines, three lines (SMG 12, SMG 21, andSMG 22) showed lower values of PHI, indicating to some extent the “lazy pod syndrome” [9,12,19,23,43].

Four lines (GGR 147, GGR 150, SMG 27, and SMG 29) stood out for maintaining PHI values greater than 60%, this being one of the main physiological traits of importance on photoassimilate remobilization toward better grain filling [12,14,15,44]. This trait has been used as a selection strategy to improve common bean yield under drought conditions [15], and is considered as a crucial trait for varietal development and genetic improvement of beans [19]. The value of PHI trait is confirmed by higher regression to GY then PNA or SNA have to GY (0.68 vs. 0.55 and 0.59, respectively). However, acid soil and high temperature stress significantly affected PNA and SNA of several lines. Interestingly, lines such as SMG 2, SMG 4, SMG 5, SMG 6, SMG 10, SMG 25 and SMG 31 were able to escape/adapt to high temperature conditions and acid soil stress through higher values of pollen viability. Lines that are adapted to acid soil and high temperature should exhibit higher sink strength through their greater ability to effectively mobilize photosynthates to pod formation and from pod walls to grains filling [42,45].

Grain yield values contributed to identify six significantly different genotypes (GGR 146, 147; SMG 12, 14, 21 and 27) in terms of sensitivity to stress. This means that in this study, the negative effect of environment was big and/or the selected lines are generally not well adapted to Amazon conditions. Higher correlation of GY to pollen viability and canopy biomass also support the idea of selection in a harsh-environment. Usually, PV and CB values have either a low or not significant effect on grain yield [9]. However, dry matter partitioning indices always played significant role.

### 3.2. Importance of Earliness and Sink Strength to Tolerate Acid Soil and High Temperature Stress

We found that some genotypes were smaller earlier (however, not significantly) with shorter time taken to reach flowering (DF ≤ 31: GGR 146, 147, 149; SMG 6, 9, 25, 26, 28, 30; SMR 168, 190). From these lines, only SMG 26, 28, 30, and SMR 190 also had a shorter (not significantly) number of days to physiological maturity (DMP ≤ 74). From the latter group, with four lines, we observed that this earliness negatively impacted the values of PNA and SNA (SMG 26, 28, 30, and SMR 190) suggesting that these lines have less time for photosynthate remobilization from vegetative parts to the pod wall (PPI) and to the seed (PHI, HI) [44,46]. Lines such as SMG 12, SMG 21, and GGR 147 showed better phenological plasticity [47] under stress conditions. Earliness is considered as a heat avoidance mechanism in grain legumes such as bean [48]; however, we found that Andean lines SAP 1, DAB 295, and DAA 129 that showed earliness in their DF and DMP values were not able to combine their short phenological cycle with higher grain yield production [40,49]. This observation is consistent with results from our previous evaluation [11].

It has been observed that high temperature stress during pre-flowering and flowering stages causes a malformation of pollen, which decreases its viability and generates flower abortion that later affects the ability to produce pods and grains [50,51,52,53]. In our study, the evaluated lines showed different responses to the combined stress from acid soil and high air temperature under field conditions. Lines such a SMG 4, 5, 6, 8, 11, 25, 31, and GGR 149 were obviously less affected by high night and day temperatures (24 and 34 °C, respectively), by presenting PV values higher than 70%. We hypothesize that for these lines, the sink strength was strictly limited as reflected by the lowest PHI values, which resulted in a reduced capacity to remobilize assimilates from vegetative structures to pod and grain formation as shown by lower grain yield value of 505 kg ha^−1^, which was significantly lower than the average (791 kg ha^−1^). 

On the other hand, lines such as GGR 146 and 147, and SMG 12, 14, and 21 showed PV values that were significantly lower than the average but their GY values were higher. Interestingly, both GGRs were earlier to flower; therefore, the reduction in PV values in these lines might have resulted from the earlier effects of high temperature on anther indehiscence [50], degeneration of the tapetum layer and a decrease in pollen production [51]. Earliness allowed these lines to improve heat tolerance through different avoidance mechanisms [48] such as CB accumulation combined with earlier flowering, as well as increased mobilization of photoassimilates towards grain formation, resulting in better yield mainly due to high SNA values [11,54].

### 3.3. Mechanisms of Photosynthetic Response of Bean Genotypes under Acid Soil and High Temperature Stress

We previously described how high temperatures affect phenological development as well as grain yield in bean lines and the different mechanisms that contribute to cope with high temperature stress in Amazon [9,11,31]. In the present study, we observed different physiological mechanisms mainly related to the functioning of the photosynthetic apparatus, measured by device MultispeQ. For example, some lines (SMG 12 and SMG 21) were able to acclimate their photosynthetic machinery to process the energy, directing it in a higher proportion in the ΦII pathway. At the same time, by generating a directionality in the use of energy, the rate of photorespiration increased during moderate or mild stress on these lines [30,55], eliminating electron pressure and allowing PSII to work more efficiently [56,57], by presenting a greater proportion of open reaction centers [30,58].

Similarly, two lines (SMG 12 and SMG 21) showed adaptive physiological mechanisms to the heat stress condition. Based on the data obtained from the correlation of the different physiological and environmental variables, we found that the decrease in LEF and ECSt (ATP synthase capacity), influenced LTD value and this could be attributed to a slight increase in abscisic acid (ABA) level leading to a higher stomatal closure [36]. These traits could be considered adaptive as they are consistent with down-regulation of ATP synthase activity to cope with low proton availability within the thylakoid membrane (gH+) [59]. Likewise, these lines increased their ΦII, which includes an activation of unregulated non-photochemical losses (ΦNO) [60,61], which could be related to smaller changes in LTD, probably as a plant protection mechanism under heat stress to preserve photochemistry [36]. 

The less negative LTD (closer to zero) in some genotypes is likely connected to lower transpiration rate [32]. Since the study was conducted under no limitation of water supply, genotypes showing less negative LTD (closer to zero) DAA 129, GGR 147, 148, 149, SMG 11 and 12 seem to be heat resistant genotypes, probably with lower water consumption (warmer leaves) and are likely to be effective in water use (e.g., SMG 12 and GGR 147 that showed higher GY values). However, further research is needed under field conditions to characterize genotypic differences in water use and transpiration efficiency.

We hypothesize that under combined acid soil and high temperature stress, bean lines activated different mechanisms to dissipate heat and keep photosystems unaffected or efficiently acclimatized by increasing transpirational flow [31,32,36]. For example, lines such as SMG 12, GGR 147 and SMG 14 presented an LTD value around −3 °C, which was lower (less negative) than that presented for genotypes SMG 23, 24, and 27 which showed an LTD value around −6 °C. This almost 3 °C difference is related to water use and transpiration efficiency. This is because in order to reduce the effect of ambient temperature on leaf temperature, transpiration must increase and, as a consequence, water use efficiency will be reduced [31]. The differences in actual leaf temperature in relation to the dissipation mechanism allowed these lines (SMG 12 and GGR 147) to have a lower cost in cooling (in terms of water movement through the plant). We can only speculate that either this saved energy resulted in a greater capacity to translocate photoassimilates for grain filling or GY was almost an independent variable. These two lines also had greater efficiency of PSII (ΦII) to regulate excess energy and carry it along the electron transport chain (LEF). Based on these characteristics, these two lines can be considered heat tolerant.

Based on different mechanisms observed in this study, we highlight the adaptive responses of lines SMG 12, SMG 14, SMG 21, SMG 27, and GGR 147 to the combined stress of acid soil and high temperature. These lines may be well adapted to higher temperature environments and may also be considered as progenitors in breeding programs focused especially on the selection of materials that combine a better physiological response in relation to carbon fixation and with a greater capacity for dry matter partitioning towards pod development and grain filling under heat stress conditions. 

### 3.4. Identification of Lines with Greater Ability in Energy Use, Leaf Cooling and Grain Yield under Acid Soil and High Temperature Stress 

The adaptation of individual genotype depends on its responses and degree of susceptibility to a stress condition. In this study, we observed that some lines have the characteristic of producing abundant biomass but with low grain yield [39,62]. Correlation analyses from this study on multiple traits indicated a positive and significant relationship between GY and the other plant traits (CB, PPI, PHI, HI, PNA, SNA and PV). This clearly indicates the importance of dry matter partitioning indices (related to photosynthate remobilization) to pod formation and grain filling under conditions of acid soil and heat stress.

In this study, we found that genotypes that tend to form larger seeds show greater sensitivity to stress, mainly in their capacity to remobilize assimilated CO_2_ for grain formation and filling. This behavior has been previously observed by Polania et al. [14] in genotypes from the Andean gene pool. In that study, Andean genotypes with greater adaptation to acid soils and high temperatures surprisingly have the same carbon accumulation potential as Mesoamerican gene pool genotypes in terms of canopy biomass. However, the smaller capacity/ability to translocate assimilates to the reproductive buds/pods (likely by greater demand between the source and the sink), translated into less effective seed production.

We highlight the high efficiency for grain formation in lines such as SMG 12 and SMG 21 that tend to be superior to lines such as SMR 180 and BFS 81 (SMG parental lines). Thus, the variables of GY, PHI and PPI were potential contributors to phenotypic differentiation at the genotype level in the common bean accessions evaluated in this study. 

The main characteristic evaluated in this study is related to the medium and large grain size of the Mesoamerican gene pool. In addition to the above, there are other characteristics that these lines have which are related to high mineral content as well as tolerance to drought and heat. For example, the GGR lines were selected based on 100-seed weight that exceeds 36 g and the GGR 146 (GGB 13 × SMR 191) line had within its progeny materials of large grains (GGB) with resistance to drought with high mineral content (SMR) while the GGR 147 (SMC 214 × (GGR 1 × SMC 232)F1) had in its cross a 75% contribution in drought resistance and mineral traits and the remaining in the size of the grain. The SMR (SMR 189 × (SMC 199 × BFS 81)) line is from gamete selection with resistance to angular spot, and tolerant to soils with low fertility and drought conditions. It was derived from a triple cross where SMR 189 provides high mineral content and adaptation to drought and good plant growth, while SMC 199 is a line that provides resistance to drought and high mineral content and good plant growth; finally, BFS 81 with tolerance to low fertility and drought. The BFS 81 line contributes 25% of the cross in terms of tolerance to acid soils and low fertility in the Amazon, a condition that has been previously reported by Suárez et al. [9,31]. Finally, the SMR line is developed from materials that have similar traits such as drought resistance, high mineral content and medium to large grains of commercial color.

Based on the results from this study, three lines (GGR 147, SMG 21 and SMG 12) were identified and these three lines combined the desirable attributes for genetic improvement of stress tolerance and biofortification in common bean. These three lines can serve as parents to further improve traits (energy use efficiency and multiple stress resistance) that are important for bean production in the Amazon region.

## 4. Materials and Methods

### 4.1. Study Area and Climatic Aonditions 

Two field trials were conducted at the Macagual Research Center of the University of Amazonia in Florencia, Colombia, located at 1°37′ N latitude and 75°36′ W longitude and an altitude of 250 meters above sea level. The first trial was conducted during December 2019 to February 2020, and the second trial was conducted during June to August 2020. The site has a tropical rainforest climate, with an annual rainfall of 3800 mm and 1700 h of sunshine per year^−1^. The average temperature is 25.5 °C and relative humidity is 84%. During the growing season the maximum and minimum air temperatures were 36.8 °C and 17.4 °C (season 1), 36.6 °C and 18.4 °C (season 2), respectively (Figure 1). The total rainfall during crop growth was 705.8 mm (season 1) and 866.4 mm (with a higher amount of precipitation before flowering; season 2) (Figure S1). Soil samples were collected at 20 cm depth and were pooled and analyzed to quantify physicochemical characteristics. The soil type is clay loam with bulk density values ranging from 1 to 1.3 g cm^−3^, pH values ranging from pH 4.1 to 5.2, with an average organic carbon content of 1.35%, available P content (Bray- II) of 2.58 mg kg^−1^, total base saturation of 7.1% (cmol(-) kg^−^^1^ : Ca, 0.38; Mg, 0.1; K, 0.14; Na, 0.1; total bases: 0.8), a cation exchange capacity of 11.3 cmol(-) kg^−^^1^, and an exchangeable Al content of 6.3 cmol(-) kg^−^^1^ with 73.4% Al saturation.

### 4.2. Plant Material and Experimental Design

For the study, 41 bean genotypes belonging to the Mesoamerican and Andean gene pool were selected: 27 advanced common bean lines interspecific mesoamerican common bean lines (SMG 1, SMG 2, SMG 3, SMG 4, SMG 5, SMG 6, SMG 7, SMG 8, SMG 9, SMG 10, SMG 11, SMG 12, SMG 13, SMG 14, SMG 19, SMG 20, SMG 21, SMG 22, SMG 23, SMG 24, SMG 25, SMG 26, SMG 27, SMG 28, SMG 29, SMG 30, and SMG 31); four advanced biofortified lines (SMR 168, SMR 169, SMR 190, SMR 191); three advanced common bean lines from the Andean gene pool (DAB 295, SAP 1 and DAA 129); seven large seeded lines (GGR 145, GGR 146, GGR 147, GGR 148, GGR 149, GGR 150, GGR 41). DAB 295 was used as a control genotype. All lines were provided by CIAT and the main characteristics of the lines are shown in Table 3. Most of these lines constitute significant advances in breeding using interspecific crosses for improving seed size, color, quality (micronutrient content) and market price combined with superior agronomic performance and multiple stress resistance. The GGR (red, cream, pink) lines are large seeded; SMG (pink, red pink mottled) and SMR (red) lines are drought resistant, with high Fe content. The DAB (red) and SAP (speckled red) lines of the Andean gene pool are better adapted to drought and are more tolerant to high temperature. The DAA (Red) line is Drought-adapted Andean bean for Africa. A randomized block design was used. Each block contained 41 genotypes. Each experimental unit consisted of 3 rows of 2 m length with a distance between rows of 0.6 m and a plant to plant spacing of 15 cm (equivalent to 11 plants m^−2^).

### 4.3. Canopy Biomass, Dry Matter Partitioning Indices, Grain Yield, and Yield Components

During the mid-pod filling growth stage (55 days after sowing in season 1, 57 days after sowing in season 2), a destructive sample was collected from a 0.5 m row segment (equivalent to an area of 0.3 m^2^) per plot. A total of four plants were sampled to determine canopy biomass (CB). Similarly, at the time of harvest, another destructive sampling was performed (0.5 m row segment, four plants), and the dry weight of leaves, stems, pods and seeds was recorded. The dry weights of the samples from two samplings, the number of seeds per area (SNA), and the number of pods per area (PNA) were determined. The following dry matter partitioning indices were determined according to Beebe et al. (2013). Pod partitioning index (PPI): dry weight of pod biomass at harvest/total canopy biomass (CB) dry weight at mid-filling × 100; pod harvest index (PHI): seed biomass dry weight at harvest/pod biomass dry weight at harvest × 100; and harvest index (HI): seed biomass dry weight at harvest/total canopy biomass dry weight at mid-pod filling × 100. We estimated HI and PPI using the CB value at mid-pod filling growth stage assuming that this is the time that reflects the maximum shoot vigor of the genotype [14]. Destructive sampling was carried out in the central part of each plot at harvest time. The pods of the harvested plants were threshed, and the grains were cleaned and dried to determine grain yield (kg ha^−1^). The weight (g) of 100 seeds was quantified with a homogeneous random sample. 

### 4.4. Phenological Responses to High Temperatures

Days to flowering (DF) was recorded for each genotype, this being the number of days after planting in which 50% of the plants have developed at least one flower. Likewise, the number of days to physiological maturity (DPM) was determined for each genotype, being the number of days after planting in which 50% of the plants had at least one pod that lost its green pigmentation. To determine the influence of high temperature on the different bean lines, pollen viability (PV) was evaluated following the methodology of Porch and Jahn [50] and Suzuki et al. [51]. Flower buds (n = 10) were collected prior to anthesis and stored in plastic jars with a 96% glacial acetic acid with alcohol solution at 4 °C. Pollen grains were removed from the anthers, and then a drop of 1% acetocarmine was added. About 100 grains of pollen from each flower bud per replicate were analyzed to determine the viability of pollen. Pollen grains that were stained red were considered viable (fertile pollen), while those that were unstained were considered infertile or non-viable. After counting, the percentage of viability was calculated as the ratio of stained grains to total grains.

### 4.5. Measurements of Photosynthetic Energy Use and Leaf Cooling under High Temperature Stress

Data related to environmental variables and photosynthetic traits based on chlorophyll fluorescence were collected using the default protocol Photosynthesis RIDES in which the measurement starts automatically once the clamp is open and closed. This protocol was developed by PhotosynQ for MultispeQ hand-held device [35]. We used the protocol followed by Deva et al. [32] for leaf sampling, and measurements were performed between 08:00 and 10:00 h (solar time) on three fully developed leaves (located between the seventh and ninth leaf developed from the base of the plant) of each plant with three independent replicates per genotype, during the flowering period (R6) which corresponds to the time when 50% of the flowers are open, 40 days after sowing, according to the BBCH scale for bean growth (BBCH 65).

Among the environmental variables, humidity and ambient temperature were measured, and the difference between the latter and the leaf temperature was used to calculate the leaf temperature differential (LTD). The linear electron transport (LEF) was estimated using the equation LEF = Φ(PAR)-YϕII, where f is the fraction (f = 0.45) of absorbed radiation which is transferred to the PSII centers and ϕII corresponds to the effective quantum yield [35]. LEF is an indicator of photosynthetic activity because it quantifies the magnitude of energy that is moved through the chloroplast when exposed to light. 

The proton conductivity of the thylakoid membrane (gH+) and the amplitude of electrochromic bandshift signal (ESCt) were determined by the dark-interval relaxation kinetic of electrochromic shift (DIRK method [63]). The measurement of gH+ reflects the activity of ATP synthase [34] and ESCt estimates the transport of protons in the transthylacoid membrane in relation to proton motive force (pmf). Based on the above, the proton flux was estimated as follows (νH+ = ECSt × gH+ [33]). 

We measured relative chlorophyll content (SPAD), the quantum efficiency for photosystem II photochemistry (ΦII), photoprotective non-photochemical quenching (ΦNPQ), and the basal dissipation of light energy (ΦNO) using the MultispeQ [35]. ΦII indicates the amount of energy received by photosystem II, it being the fluorescence parameter indicating the photosynthetic state of the plant. ΦNPQ is the energy dissipated as heat and ΦNO indicates the energy that has not been used by the leaf for photosynthesis or dissipated and represents potential damage. ΦNPQ and ΦNO were calculated by applying the equations derived by Kramer et al. [64]. The summation of ΦII, ΦNPQ, and ΦNO is equal to one. 

The quenching due to non-photochemical dissipation of absorbed light energy (NPQt) was calculated according to Tietz et al. [65]. Likewise, different PSI redox states were estimated from those described by Kanazawa et al. [34], which corresponded to the total active PSI centers (PSIact), the fraction of oxidized PSI centers (PSIox) and open state (PSIopen) as well as over-reduced PSI (PSIor) corresponding to PSI acceptor side limitations resulting from the accumulation of electrons in the PSI acceptors during steady-state illumination. 

According to the information reported in the PhotosynQ platform, the total number of data obtained by the MultispeQ device during sampling was 878, in which there were no errors. Fluorescence outliers were identified and excluded from the analysis using Pearson’s standardized residuals. This type of residual provides a measure of how well the observation is predicted by the model, by recording those data that do not present an adequate fit [66].

### 4.6. Data Analysis

To determine the differences between bean lines in relation to the different variables of agronomic performance and chlorophyll fluorescence, a Generalized Linear Model (GLM) was fitted. The bean line was selected as a fixed factor and the blocks nested within the growth stage of evaluation in each season and the plots associated with the bean lines within the blocks were included as random effects. The assumptions of normality and homogeneity of variance were evaluated using an exploratory analysis of residuals. To determine the differences between evaluated bean lines, Fisher’s post hoc LSD test was used with a significance of α = 0.05. The association between agronomic, phenological, environmental and chlorophyll fluorescence-related variables was analyzed by calculating Pearson’s correlation coefficients. Different scatter plots were made to determine the lines with the best adaptation to acid soils and high temperature stress. On the Y axis of the scatter plots were placed variables of agronomic performance such as grain yield as well as variables related to energy use such as ΦII, ΦNPQ, and ΦNO and those affecting these variables were plotted on the X axis which corresponded to CB, PPI, PHI, ΦII, LEF and LTD. The variables located in the scatter plots were associated with the effect of the gradient of different variables. Their intensity is identified with the change in the color (of violet and red from higher to lower value) and as well as the magnitude that related to circle size. The graphs were visualized in four quadrants by plotting the corresponding means on each axis. For the Generalized Linear Model (GLM) analysis, we used the lme function of the nlme package [67], both GLM and Principal Component Analysis (PCA) were performed in R language software, version R.4.1.0 [68] and we used the interface in InfoStat [69]. The scatter plots were made using the ggplot2 package [70] in R language software, version R.4.1.0.

## 5. Conclusions

Based on physiological evaluation of the energy use, leaf cooling, photosynthate partitioning for pod formation, filling and grain yield of the different bean lines, we found that three bean lines (GGR 147, SMG 21 and SMG 12) were superior in their photosynthetic response resulting in grain yields that were above 1900 kg ha^−1^ under acid soil stress and high temperature conditions. The heat stress tolerance of the different lines evaluated was attributed to the efficient use of the energy absorbed at the electron level in the thylakoids, which was mainly oriented to a higher quantum yield of PSII (ΦII), coupled with a lower dissipation of energy as heat (ΦNPQ), as well as a high linear electron flux (LEF) and a high fraction of PSI centers in the open state (PSIopen). These physiological changes translated into the ability to produce greater canopy biomass, phenological adjustments in relation to days to flowering and maturity as well as the efficiency in mobilizing photoassimilates to produce more pods and grains. These three (GGR 147, SMG 21 and SMG 12) physiologically efficient lines combined desirable attributes for genetic improvement of stress tolerance and biofortification and, therefore, can serve as parents to further improve the traits (energy use efficiency and resistance to multiple stresses) that are important for bean production in the Amazon region.

## Figures and Tables

**Table 1:** Correlation coefficients (r) between grain yield, pollen viability , pod harvest index, canopy biomass, leaf temperature differential and other plant attributes of 41 bean genotypes grown under conditions of acid soil and high temperature stress.

Variables	Grain Yield (kg ha^−1^)	Pollen Viability (%)	Pod Harvest Index (PHI, %)	Canopy Biomass (CB, kg ha^−1^)
Grain yield (kg ha^−1^)		−0.47 **	0.68 ***	0.33 *
Canopy biomass (CB, kg ha^−1^)	0.33 *	−0.2	−0.1	
Pod partitioning index (PPI,%)	0.74 ***	−0.36 *	0.68 ***	−0.01
Pod harvest index (PHI, %)	0.68 ***	−0.27		−0.1
Harvest index (HI, %)	0.06	0.02	0.38 *	−0.08
Pod number per area (PNA)	0.92 ***	−0.42 **	0.55 **	0.3
Seed number per area (SNA)	0.96 ***	−0.43 **	0.59 **	0.35 *
Days to flowering	0.07	0.14	0.08	−0.06
Days to maturity	0.02	−0.03	−0.02	−0.17
Pollen viability (%)	−0.47 **		−0.27	−0.2

Mean values were used in the correlation analysis and *, **, and *** represent levels of significance at 0.05, 0.01, and 0.001, respectively.

**Table 2:** Correlation coefficients (*p* < 0.05) between different variables related to photosynthetic apparatus functioning and dry matter partitioning indices, phenology and leaf temperature differential of 41 bean genotypes grown under acid soil and high temperature stress.

Variables	Canopy Biomass (CB, kg ha^−1^)	Harvest Index (HI, %)	Pod Harvest Index (PHI, %)	Seed Number Per Area (SNA)	Days to Flowering (DF)	Days to Maturity (DMP)	Pollen Viability (PV %)	Leaf Temperature Differential (LTD)
LTD				0.28		0.38	−0.3	
gH+								−0.30
vH+	−0.27	0.34						
ECSt		0.31						0.31
LEF		0.31					0.27	
ΦII		0.33	0.31					−0.34
ΦNPQ	−0.30		−0.32					0.31
ΦNO	0.30					0.35		0.34
NPQt	−0.29		−0.36			−0.32		
PSIact	0.31				0.32	−0.42	0.28	
PSIor		0.34					−0.28	
PSIox	0.27	−0.38					0.29	
SPAD						0.27		

**Table 3:** List of interspecific Mesoamerican common bean lines to acid soils and high temperature stress at the Macagual Research Center of the University of Amazonia during two seasons (2019, 2020).

Genotype.	Commercial Class	Gene Pool	Growth Habit	Cross
DAA 129	Red pink	Andean	II A	Pv
DAB 295	Red mottled	Andean	I	Pv
GGR 145	Red	Mesoamerican	II B	Pv
GGR 146	Cream red mottled	Mesoamerican	II B	Pv
GGR 147	Cream red mottled	Mesoamerican	II B	Pv
GGR 148	Red	Mesoamerican	II B	Pv
GGR 149	Red Pink mottled	Mesoamerican	II B	Pv × Pc × Pa × Pd
GGR 150	Cream red mottled	Mesoamerican	II B	Pv × Pc × Pa × Pd
GGR 41	Cream	Mesoamerican	II A	Pv
SAP 1	Red mottled	Andean	I	Pv
SMG 1	Red	Mesoamerican	II B	Pv × Pc × Pa × Pd
SMG 2	Pink	Mesoamerican	II B	Pv × Pc × Pa × Pd
SMG 3	Pink	Mesoamerican	II B	Pv × Pc × Pa × Pd
SMG 4	Pink	Mesoamerican	II B	Pv × Pc × Pa × Pd
SMG 5	Pink	Mesoamerican	II B	Pv × Pc × Pa × Pd
SMG 6	Pink	Mesoamerican	II A	Pv × Pc × Pa × Pd
SMG 7	Pink	Mesoamerican	II A	Pv × Pc × Pa × Pd
SMG 8	Pink	Mesoamerican	II A	Pv × Pc × Pa × Pd
SMG 9	Pink	Mesoamerican	II A	Pv × Pc × Pa × Pd
SMG 10	Pink	Mesoamerican	II B	Pv × Pc × Pa × Pd
SMG 11	Pink	Mesoamerican	II B	Pv × Pc × Pa × Pd
SMG 12	Pink	Mesoamerican	II A	Pv × Pc × Pa × Pd
SMG 13	Pink	Mesoamerican	II A	Pv × Pc × Pa × Pd
SMG 14	Pink	Mesoamerican	II A	Pv × Pc × Pa × Pd
SMG 19	Red Pink mottled	Mesoamerican	II A	Pv × Pc × Pa × Pd
SMG 20	Red Pink mottled	Mesoamerican	II B	Pv × Pc × Pa × Pd
SMG 21	Red Pink mottled	Mesoamerican	II B	Pv × Pc × Pa × Pd
SMG 22	Red Pink mottled	Mesoamerican	II B	Pv × Pc × Pa × Pd
SMG 23	Red Pink mottled	Mesoamerican	II B	Pv × Pc × Pa × Pd
SMG 24	Red Pink mottled	Mesoamerican	II B	Pv × Pc × Pa × Pd
SMG 25	Red Pink mottled	Mesoamerican	II B	Pv × Pc × Pa × Pd
SMG 26	Red Pink mottled	Mesoamerican	II B	Pv × Pc × Pa × Pd
SMG 27	Red Pink mottled	Mesoamerican	II B	Pv × Pc × Pa × Pd
SMG 28	Red Pink mottled	Mesoamerican	II B	Pv × Pc × Pa × Pd
SMG 29	Red Pink mottled	Mesoamerican	II B	Pv × Pc × Pa × Pd
SMG 30	Red Pink mottled	Mesoamerican	II B	Pv × Pc × Pa × Pd
SMG 31	Red Pink mottled	Mesoamerican	II B	Pv × Pc × Pa × Pd
SMR 168	Red	Mesoamerican	II B	Pv
SMR 169	Red	Mesoamerican	II B	Pv
SMR 190	Red	Mesoamerican	II B	Pv
SMR 191	Red	Mesoamerican	II B	Pv

Pv: *Phaseolus vulgaris*, Pc: *Phaseolus coccineus*, Pa: *Phaseolus acutifolius*, Pd: *Phaseolus dumosus*. Growth habit type I is an erect determinate bush, growth habit type IIA is shrub with short guide, growth habit type IIB is an semierect indeterminate.

## Data Availability

Data are available from the authors upon request.

## Supplementary Materials

The following are available online at https://www.mdpi.com/article/10.3390/plants10112412/s1, Table S1: Genotypic differences in canopy biomass (CB), pod partitioning index (PPI), pod harvest index (PHI), harvest index (HI), pod number per area (PNA), seed number per area (SNA), grain yield (GY), 100 seeds weight (SW), days to flowering (DF), days to physiological maturity (DPM) and pollen viability (PV) of 41 bean genotypes grown under conditions of acid soil and high temperature stress, Table S2: Physiological differences in Leaf Temperature Differential (LTD), relative chlorophyll content (SPAD), linear electron transport (LEF), proton conductivity of the thylakoid membrane (gH+), amplitude of electrochromic bandshift signal (ESCt), proton flux (νH+), photosystem II photochemistry (ΦII), photoprotective non-photochemical quenching (ΦNPQ), basal dissipation of light energy (ΦNO), quenching due to non-photochemical dissipation of absorbed light energy (NPQt), total active PSI centers (PSIact), fraction of oxidized PSI centers (PSIox) pen state (PSIopen) as well as over-reduced PSI (PSIor) of 41 bean genotypes grown under conditions of acid soil and high temperature stress, Figure S1 Distribution of rainfall and maximum/minimum temperatures during the growing season at the Macagual Research Center in Colombia in two seasons: December 2019–March 2020 (left); and June–September 2020 (right).
