# Supplementary material for: Adaptation of Interspecific Mesoamerican Common Bean Lines to Acid Soils and High Temperature in the Amazon Region of Colombia
Source: Plants (Basel). 2021 Nov 9;10(11):2412. doi: 10.3390/plants10112412 (PMC8623317; doi:10.3390/plants10112412)
Supplement: Supplementary file 1 [file plants-10-02412-s001.zip › plants-1425338-proofed suppl/Supplementary material 1.pdf]

Supplementary material 1. Genotypic differences in canopy biomass (CB), pod partitioning index (PPI), pod harvest index (PHI), harvest index (HI), pod number per area (PNA), seed number per area (SNA), grain yield (GY), 100 seeds weight (SW), days to flowering (DF), days to physiological maturity (DPM) and viability of pollen (VP) of 41 bean genotypes grown under conditions of acid soil and high temperature stress.

Genotipo	CB	PPI	PHI	HI	PNA	SNA	GY	SW	DF	DPM	VP
DAA 129	1805.97 ± 220.24 b	65.64 ± 3.09 a	20.02 ± 3.86 b					26.5 ± 1.9 b	32 ± 1.31 a	76 ± 2.17 a	36.98 ± 3.94 c
DAB 295	1526.53 ± 220.24 b	47.34 ± 13.83 b		23.98 ± 5.37 a	86.68 ± 17.08 c	229.42 ± 55.7 c	762.26 ± 163.32 b	31.46 ± 0.91 a	32.46 ± 1.31 a	75 ± 1.82 a	35.99 ± 3.94 c
GGR 145	1746.39 ± 220.24 b							25.73 ± 3.29 b	32.46 ± 1.31 a		
GGR 146	1585.28 ± 310.44 b	65.41 ± 3.41 a			124.74 ± 22.02 c	444.98 ± 108 c	1494.02 ± 228.24 a		30.96 ± 1.31 a	76 ± 1.82 a	43.3 ± 3.94 c
GGR 147	1680.58 ± 253.88 b		79.9 ± 10.94 a	27.99 ± 10.72 a	178.38 ± 29.3 b	598.9 ± 145.4 b	1922.55 ± 320.66 a	31.12 ± 1.55 a	30.96 ± 1.31 a	79 ± 1.82 a	47.36 ± 3.94 c
GGR 148	1745.73 ± 277.88 b	55.58 ± 7.58 a	21.08 ± 10.98 b	42.38 ± 15.16 a	60.99 ± 40 c	172.79 ± 41.9 c	487.79 ± 451.37 b	26.17 ± 1.98 b	33.96 ± 1.31 a	78 ± 1.82 a	63.24 ± 3.94 b
GGR 149	1408.89 ± 310.49 b	38.18 ± 4.84 b	14.01 ± 3.12 b	30.19 ± 7.59 a	54.18 ± 22.09 c	159.96 ± 38.8 c	490.51 ± 228.57 b	25.42 ± 1.6 b	29.35 ± 1.31 a	75 ± 1.82 a	72.01 ± 3.94 b
GGR 150	1383.36 ± 188.05 b	42.62 ± 17.46 b	61.39 ± 6.81 a	42.46 ± 6.2 a	89.58 ± 17.04 c	241.87 ± 58.7 c	840.41 ± 163.1 b	30.87 ± 0.85 a	34 ± 1.07 a	71 ± 1.82 a	56.35 ± 3.94 b
GGR 41		23.17 ± 4.3 b			112.58 ± 29.25 c	256.43 ± 62.2 c	627.38 ± 320.45 b	23.37 ± 2.32 b		78 ± 1.82 a	67.15 ± 3.94 b
SAP 1	994.97 ± 220.11 b						±	23.92 ± 1.55 b	33.61 ± 1.83 a	78 ± 1.82 a	60.69 ± 3.94 b
SMG 1	1579.39 ± 235.26 b	19.02 ± 3.18 b	2.09 ± 0.23 c	16.16 ± 10.72 a	26.01 ± 24.73 c	60.69 ± 14.7 c	157.08 ± 262.91 b	22.47 ± 1.9 b	32.46 ± 1.31 a	76 ± 1.82 a	48.23 ± 3.94 c
SMG 2	1964.86 ± 220.24 b	21.98 ± 4.47 b	8.37 ± 1.95 b	24.64 ± 7.59 a	54.74 ± 17.08 c	125.16 ± 30.4 c	361.94 ± 163.32 b	22.45 ± 1.34 b	32.46 ± 1.31 a	75 ± 1.82 a	78.83 ± 3.94 a
SMG 3	1286.13 ± 235.17 b									78 1.82 a	58.4 3.94 b
SMG 4	1541.63 ± 220.11 b	30.6 ± 14.13 b	18.13 ± 7.3 b	51.89 ± 8.76 a	19.18 ± 22.02 c	56.27 ± 13.7 c	228.69 ± 262.63 b	26.78 ± 1.55 b	33.96 ± 1.31 a	75 ± 1.82 a	74.77 ± 3.94 b
SMG 5	1158.41 ± 235.17 b	33.69 ± 3.33 b	13.13 ± 10.94 b	40.45 ± 15.17 a	80.7 ± 24.85 c	158.7 ± 38.5 c	435.94 ± 264.09 b	22.61 ± 1.7 b	34 ± 1.07 a	75 ± 1.82 a	72.39 ± 3.94 b
SMG 6	1186.67 ± 220.24 b	66.82 ± 9.78 a	15.21 ± 7.94 b	15.18 ± 7.58 a	51.96 ± 17.08 c	117.34 ± 28.5 c	391.65 ± 163.32 b	27.49 ± 1.6 b	31 ± 1.07 a	75 ± 1.82 a	71.74 ± 3.94 b
SMG 7	1641.11 ± 310.44 b					±	±	25.28 ± 3.29 b	34.31 ± 1.83 a	79 ± 1.82 a	62.39 ± 3.94 b
SMG 8	1587.47 ± 358.16 b	26.34 ± 4.37 b	3.15 ± 0.03 c	13.83 ± 8.76 a	44.53 ± 24.73 c	101.78 ± 24.7 c	285.21 ± 262.91 b	25.78 ± 3.29 b	33.96 ± 1.31 a	76 ± 1.82 a	82.28 ± 3.94 a
SMG 9	1509.08 ± 235.26 b	25.8 ± 7.51 b	4.96 ± 2.1 c	27.5 ± 7.59 a	39.57 ± 17.84 c	108.86 ± 26.4 c	335.32 ± 174.08 b	26.89 ± 1.44 b	30.96 ± 1.31 a	76 ± 1.82 a	62.28 ± 3.94 b
SMG 10	1683.76 ± 220.24 b	21.14 ± 6.08 b	4.3 ± 2.03 c	39.52 ± 10.72 a	26.4 ± 22.09 c	64.91 ± 15.8 c	186.9 ± 228.57 b	23.26 ± 4.65 b	33 ± 1.07 a	76 ± 1.82 a	81.5 ± 3.94 a
SMG 11	1199.33 ± 253.85 b							22.69 ± 1.7 b	34.31 ± 1.83 a	76 ± 1.82 a	70.07 ± 3.94 b
SMG 12	2009.76 ± 235.24 b	65.78 ± 27.35 a	37.72 ± 3.22 b	29.55 ± 7.59 a	268.07 ± 22.09 a	886.08 ± 215.1 a	2152.75 ± 320.62 a	28.61 ± 0.92 b	32.46 ± 1.31 a	78 ± 1.82 a	58.02 ± 3.94 b
SMG 13	1446.5 ± 220.11 b	17.22 ± 4.97 b	2.96 ± 2.01 c	26.22 ± 10.73 a	51.9 ± 17.79 c	118.37 ± 28.7 c	371.54 ± 173.96 b	21.3 ± 3.29 b	32.46 ± 1.31 a	75 ± 1.82 a	54.83 ± 3.94 b
SMG 14	2013.06 ± 220.24 b	48.82 ± 11 b	25.88 ± 6.61 b	31.79 ± 7.59 a	148.63 ± 22.09 b	477.06 ± 115.8 b	1599.52 ± 228.57 a	27.47 ± 1.05 b	33.96 ± 1.31 a	76 ± 1.82 a	46.23 ± 3.94 c
SMG 19	1309.36 ± 180.17 b	58.96 ± 7.37 a	34.76 ± 5.7 b	39.35 ± 5.37 a	86.11 ± 14.98 c	256.53 ± 62.3 c	736.47 ± 134.37 b	24.05 ± 0.84 b	34 ± 1.07 a	74 ± 1.82 a	42.48 ± 3.94 c
SMG 20	1480.91 ± 188.11 b	52.49 ± 9.29 b	22.01 ± 4.71 b	42.94 ± 4.58 a	59.26 ± 14.98 c	162.37 ± 39.4 c	625.11 ± 140.06 b	27.85 ± 0.75 b	33 ± 1.07 a	76 ± 1.82 a	48.53 ± 3.94 c
SMG 21	2295.76 ± 180.17 a	72.13 ± 10.05 a	31.15 ± 3.79 b	29.23 ± 4.38 a	169.44 ± 14.98 b	553.38 ± 134.3 b	2007.07 ± 134.37 a	28.25 ± 0.49 b	34 ± 1.07 a	71 ± 1.82 a	44.82 ± 3.94 c
SMG 22	1217.57 ± 180.17 b	60.43 ± 11.18 a	38.65 ± 10.9 b	40.75 ± 6.2 a	90.47 ± 18.84 c	248.67 ± 60.4 c	951.23 ± 187.53 b	28.62 ± 0.84 b	34 ± 1.31 a	72 ± 1.82 a	67.72 ± 3.94 b
SMG 23	2179.72 ± 220.24 a	41.47 ± 6.32 b	15.31 ± 6.42 b	32.33 ± 7.58 a	68.63 ± 17.08 c	218.99 ± 53.2 c	747.51 ± 163.32 b	24.18 ± 0.87 b	34 ± 1.31 a	71 ± 1.82 a	60.3 ± 3.98 b

SMG 24	1462.48	±	180.17	b	50.6	±	10.22	b	26.57	±	4.67	b	37.87	±	4.38	a	78.7	±	14.98	c	247.34	±	60	c	917.58	±	134.37	b	28.44	±	0.72	b	34	±	1.07	a	71	±	1.82	a	61.92	±	3.94	b
SMG 25	1364.85	±	188.06	b	32.86	±	6.35	b	18.18	±	4.2	b	34.93	±	5.06	a	55.07	±	15.35	c	159.29	±	38.7	c	502.62	±	140.01	b	26.84	±	1.04	b	31	±	1.83	a	76	±	1.82	a	78.61	±	3.94	a
SMG 26	1029.64	±	207.71	b	56.55	±	6.82	a	35.29	±	6.92	b	41.67	±	5.37	a	76.96	±	17.08	c	241.01	±	58.5	c	878.32	±	163.32	b	29.73	±	0.81	a	31	±	1.07	a	72	±	1.82	a	51.41	±	3.94	c
SMG 27	1623.19	±	220.24	b	78.3	±	14.91	a	63.84	±	14.51	a	42.77	±	10.72	a	117.27	±	29.3	c	426.04	±	103.4	c	1630.65	±	320.66	a	31.3	±	1.16	a	34	±	1.31	a	74	±	1.82	a	67.17	±	3.94	b
SMG 28	1943.2	±	235.26	b	45.99	±	7.88	b	33.71	±	7.74	b	51.94	±	7.59	a	66.55	±	17.84	c	215.95	±	52.4	c	813.79	±	174.08	b	28.73	±	1.03	b	29	±	1.31	a	72	±	1.82	a	63.8	±	3.94	b
SMG 29	1372.06	±	235.17	b	61.72	±	15.09	a	60.32	±	10.93	a	39.51	±	7.59	a	62.51	±	22.09	c	241.46	±	58.6	c	867.24	±	228.57	b	33.08	±	1.44	a	34	±	1.07	a	76	±	1.82	a	67.33	±	3.94	b
SMG 30	2319.59	±	235.17	a	40.36	±	7.18	b	18.74	±	7.12	b	33.72	±	7.59	a	52.52	±	22.02	c	189.65	±	46	c	661.1	±	228.24	b	27.39	±	0.86	b	30.96	±	1.31	a	74	±	1.82	a	69.06	±	3.94	b
SMG 31	1265.56	±	173.12	b	30.1	±	5.45	b	17.8	±	3.05	b	32	±	5.37	a	41.67	±	14.98	c	99.62	±	24.2	c	358.38	±	140.06	b	26.87	±	1.01	b	33.96	±	1.31	a	72	±	1.82	a	79.99	±	3.94	a
SMR 168	1462.78	±	310.49	b																													31.31	±	1.83	a	78	±	1.82	a	38.97	±	3.94	c
SMR 169	1790.83	±	310.44	b																									21.86	±	1.98	b					76	±	1.82	a	55.9	±	3.94	b
SMR 190	1807.97	±	235.26	b	24.85	±	14.21	b	28.91	±	12.75	b	45.45	±	5.74	a	55.44	±	17.84	c	156.63	±	38	c	528.18	±	174.08	b	26.62	±	0.87	b	29.46	±	1.31	a	74	±	1.82	a	48.15	±	3.94	c
SMR 191	2455.67	±	180.17	a	33.89	±	4.86	b	10.85	±	1.4	b	30.49	±	4.58	a	82.41	±	14.98	c	275	±	66.7	c	760.68	±	134.37	b	21.98	±	0.76	b	33.61	±	1.83	a	72	±	1.82	a	47.99	±	3.94	c
