# Supplementary material for: Adaptation of Interspecific Mesoamerican Common Bean Lines to Acid Soils and High Temperature in the Amazon Region of Colombia
Source: Plants (Basel). 2021 Nov 9;10(11):2412. doi: 10.3390/plants10112412 (PMC8623317; doi:10.3390/plants10112412)
Supplement: Supplementary file 1 [file plants-10-02412-s001.zip › plants-1425338-proofed suppl/Supplementary material 2.pdf]

Supplementary Material 2. Physiological differences in Leaf Temperature Differential (LTD), relative chlorophyll content (SPAD), linear electron transport (LEF), proton conductivity of the thylakoid membrane (gH+), amplitude of electrochromic bandshift signal (ESCt), proton flux (νH+), photosystem II photochemistry (ΦII), photoprotective non-photochemical quenching (ΦNPQ), basal dissipation of light energy (ΦNO), quenching due to non-photochemical dissipation of absorbed light energy (NPQt), total active PSI centers (PSIact), fraction of oxidized PSI centers (PSIox) pen state (PSIopen) as well as over-reduced PSI (PSIor) of 41 bean genotypes grown under conditions of acid soil and high temperature stress.

Genotipo	LTD	SPAD	LEF	gH+	ECSt	vH+	ΦII	ΦNPQ	ΦNO	NPQt	PSIact	PSIopen	PSIor	PSIox
DAA 129	-2.2 ± 0.9 a	30.8 ± 2.1	187.4 ± 26.2 b	224.0 ± 10.5	9.8 ± 1.1 b	0.21 ± 0.02 c	0.48 ± 0.02	0.30 ± 0.03	0.22 ± 0.01	1.46 ± 0.26	1.28 ± 0.17 d	0.49 ± 0.24	-0.39 ± 0.12	0.90 ± 0.27
DAB 295	-5.3 ± 0.1 b	29.0 ± 1.6	177.5 ± 16.3 b	207.8 ± 3.5	11.2 ± 1.2 a	0.23 ± 0.02 b	0.46 ± 0.03	0.34 ± 0.04	0.20 ± 0.02	1.88 ± 0.36	0.42 ± 0.48 c	0.34 ± 0.29	0.01 ± 0.84	0.65 ± 0.69
GGR 145	-3.4 ± 0.4 a	30.9 ± 1.1	194.1 ± 17.5 a	213.7 ± 6.3	11.2 ± 1.0 a	0.24 ± 0.02 b	0.48 ± 0.03	0.31 ± 0.04	0.21 ± 0.02	1.73 ± 0.42	1.09 ± 0.17 d	0.51 ± 0.23	-0.87 ± 0.44	1.36 ± 0.57
GGR 146	-3.9 ± 0.6 a	30.5 ± 1.4	167.0 ± 25.3 b	217.1 ± 5.2	10.6 ± 1.7 a	0.23 ± 0.03 b	0.47 ± 0.03	0.32 ± 0.04	0.21 ± 0.01	1.60 ± 0.23	1.36 ± 0.70 d	0.29 ± 0.15	-0.08 ± 0.31	0.79 ± 0.23
GGR 147	-3.5 ± 0.6 a	31.3 ± 1.1	226.2 ± 21.1 a	224.3 ± 5.6	11.2 ± 1.0 a	0.25 ± 0.02 b	0.46 ± 0.03	0.34 ± 0.04	0.19 ± 0.01	1.97 ± 0.37	0.39 ± 0.75 c	1.37 ± 0.64	-1.52 ± 1.30	1.14 ± 0.77
GGR 148	-1.9 ± 0.5 a	31.7 ± 0.7	200.8 ± 20.9 a	221.3 ± 3.3	11.5 ± 0.9 a	0.25 ± 0.02 b	0.47 ± 0.01	0.32 ± 0.02	0.21 ± 0.01	1.61 ± 0.20	0.98 ± 0.44 d	-0.08 ± 0.60	0.96 ± 0.82	0.12 ± 0.30
GGR 149	-3.3 ± 0.5 a	32.7 ± 1.7	200.5 ± 14.3 a	223.2 ± 3.9	11.7 ± 0.8 a	0.26 ± 0.02 a	0.47 ± 0.01	0.30 ± 0.02	0.22 ± 0.01	1.44 ± 0.20	1.30 ± 0.13 d	0.58 ± 0.12	-0.65 ± 0.28	1.07 ± 0.34
GGR 150	-4.3 ± 0.6 b	29.9 ± 1.4	203.8 ± 16.2 a	217.4 ± 4.3	11.4 ± 1.2 a	0.25 ± 0.02 b	0.46 ± 0.02	0.36 ± 0.03	0.19 ± 0.01	2.06 ± 0.35	0.32 ± 0.60 c	0.43 ± 0.19	0.64 ± 0.93	-0.07 ± 0.87
GGR 41	-4.4 ± 0.9 b	33.3 ± 0.9	177.2 ± 28.7 b	234.2 ± 11.0	9.3 ± 1.7 b	0.21 ± 0.03 c	0.50 ± 0.02	0.27 ± 0.02	0.24 ± 0.01	1.18 ± 0.15	1.10 ± 0.72 d	0.84 ± 0.17	-0.18 ± 0.38	0.34 ± 0.27
SAP 1	-5.4 ± 0.5 b	34.2 ± 1.8	216.4 ± 33.9 a	236.8 ± 5.8	9.8 ± 1.2 b	0.23 ± 0.03 b	0.49 ± 0.02	0.28 ± 0.03	0.22 ± 0.01	1.37 ± 0.22	1.86 ± 1.21 d	0.55 ± 0.15	-1.77 ± 1.85	2.21 ± 1.82
SMG 1	-5.0 ± 0.3 b	29.4 ± 1.1	146.2 ± 15.2 b	240.8 ± 12.6	8.9 ± 1.0 c	0.21 ± 0.02 c	0.51 ± 0.01	0.28 ± 0.02	0.22 ± 0.01	1.37 ± 0.23	-0.68 ± 1.83 b	0.72 ± 0.22	-0.30 ± 0.25	0.59 ± 0.13
SMG 10	-4.7 ± 0.6 b	35.5 ± 3.7	255.2 ± 30.2 a	216.9 ± 6.8	14.5 ± 1.8 a	0.31 ± 0.03 a	0.44 ± 0.02	0.36 ± 0.02	0.20 ± 0.01	1.75 ± 0.10	1.50 ± 0.24 d	0.40 ± 0.09	-0.31 ± 0.05	0.91 ± 0.10
SMG 11	-3.3 ± 0.8 a	30.8 ± 3.9	214.4 ± 23.2 a	214.9 ± 4.2	12.6 ± 1.2 a	0.27 ± 0.02 a	0.43 ± 0.03	0.34 ± 0.03	0.22 ± 0.02	1.68 ± 0.28	0.96 ± 0.85 a	1.14 ± 0.71	-0.88 ± 0.99	0.74 ± 0.29
SMG 12	-3.2 ± 1.0 a	34.4 ± 1.4	179.8 ± 14.1 b	216.8 ± 4.8	10.4 ± 0.9 a	0.22 ± 0.02 b	0.49 ± 0.02	0.28 ± 0.03	0.23 ± 0.01	1.27 ± 0.20	1.21 ± 0.13 d	0.86 ± 0.18	-0.71 ± 0.23	0.85 ± 0.19
SMG 13	-3.6 ± 0.9 a	31.6 ± 1.5	157.9 ± 16.1 b	222.5 ± 6.1	10.0 ± 1.6 a	0.22 ± 0.03 b	0.49 ± 0.03	0.30 ± 0.04	0.21 ± 0.02	1.63 ± 0.40	1.34 ± 0.20 d	0.70 ± 0.21	-0.58 ± 0.33	0.89 ± 0.27
SMG 14	-3.7 ± 0.9 a	31.6 ± 2.5	163.0 ± 35.3 b	207.6 ± 26.3	9.6 ± 2.0 b	0.19 ± 0.04 c	0.46 ± 0.04	0.33 ± 0.04	0.20 ± 0.01	1.81 ± 0.37	1.06 ± 0.51 d	1.08 ± 0.78	-0.75 ± 0.64	0.67 ± 0.33
SMG 19	-4.9 ± 0.5 b	36.7 ± 2.3	142.8 ± 17.3 b	228.8 ± 5.9	7.4 ± 0.8 c	0.17 ± 0.02 c	0.53 ± 0.01	0.25 ± 0.02	0.22 ± 0.01	1.15 ± 0.13	1.50 ± 0.19 d	0.86 ± 0.13	-0.50 ± 0.13	0.64 ± 0.16
SMG 2	-5.5 ± 0.3 b	34.0 ± 2.2	178.8 ± 20.8 b	216.5 ± 6.6	9.3 ± 1.0 b	0.20 ± 0.02 c	0.50 ± 0.02	0.28 ± 0.03	0.22 ± 0.01	1.38 ± 0.23	0.85 ± 0.23 c	0.29 ± 0.38	-0.29 ± 0.69	1.00 ± 0.37
SMG 20	-3.8 ± 0.5 a	32.4 ± 0.8	224.2 ± 20.7 a	225.3 ± 1.8	12.0 ± 0.7 a	0.27 ± 0.01 a	0.47 ± 0.01	0.31 ± 0.02	0.22 ± 0.01	1.50 ± 0.17	0.93 ± 0.28 c	0.78 ± 0.38	-1.43 ± 1.63	1.65 ± 1.26
SMG 21	-5.5 ± 0.3 b	31.9 ± 1.6	180.9 ± 19.7 b	226.2 ± 6.0	9.1 ± 0.8 b	0.20 ± 0.02 c	0.51 ± 0.01	0.27 ± 0.02	0.22 ± 0.01	1.28 ± 0.17	0.26 ± 0.78 c	0.73 ± 0.31	-0.27 ± 0.36	0.54 ± 0.18
SMG 22	-4.3 ± 0.6 b	32.4 ± 1.7	250.9 ± 23.2 a	217.9 ± 5.1	13.7 ± 1.2 a	0.30 ± 0.02 a	0.43 ± 0.03	0.39 ± 0.04	0.18 ± 0.01	2.33 ± 0.39	1.06 ± 0.44 d	0.61 ± 0.30	-0.22 ± 0.50	0.61 ± 0.26
SMG 23	-6.2 ± 0.3 b	33.2 ± 1.1	175.8 ± 14.6 b	224.6 ± 5.9	8.8 ± 0.9 c	0.20 ± 0.02 c	0.52 ± 0.01	0.26 ± 0.01	0.22 ± 0.01	1.22 ± 0.09	0.40 ± 0.76 c	0.52 ± 0.20	-0.20 ± 0.41	0.67 ± 0.33

SMG 24	-6.0 ± 0.2	b	32.5 ± 1.8	196.9 ± 10.8	a	220.9 ± 7.2	11.3 ± 0.7	a	0.25 ± 0.01	b	0.45 ± 0.02	0.32 ± 0.03	0.22 ± 0.01	1.55 ± 0.21	1.35 ± 0.15	d	0.43 ± 0.10	-0.36 ± 0.13	0.93 ± 0.13
SMG 25	-4.7 ± 0.3	b	32.2 ± 1.3	233.5 ± 19.5	a	234.1 ± 4.2	12.0 ± 0.9	a	0.28 ± 0.02	a	0.46 ± 0.01	0.34 ± 0.02	0.20 ± 0.01	1.74 ± 0.17	1.44 ± 0.18	d	0.58 ± 0.11	-0.27 ± 0.16	0.69 ± 0.18
SMG 26	-4.5 ± 0.3	b	30.7 ± 1.4	209.8 ± 16.3	a	219.2 ± 6.3	12.7 ± 1.2	a	0.27 ± 0.02	a	0.46 ± 0.03	0.33 ± 0.03	0.21 ± 0.01	1.73 ± 0.32	0.92 ± 0.50	c	0.52 ± 0.19	-0.21 ± 0.64	0.69 ± 0.61
SMG 27	-6.4 ± 0.2	b	33.6 ± 1.6	162.7 ± 20.3	b	234.4 ± 6.2	8.8 ± 1.2	c	0.20 ± 0.03	c	0.51 ± 0.01	0.25 ± 0.02	0.24 ± 0.01	1.08 ± 0.17	-0.14 ± 0.57	b	0.42 ± 0.55	-0.13 ± 0.83	0.71 ± 0.32
SMG 28	-4.7 ± 0.4	b	31.2 ± 1.7	182.6 ± 23.4	b	244.0 ± 28.6	11.1 ± 1.7	a	0.24 ± 0.03	b	0.46 ± 0.03	0.32 ± 0.03	0.21 ± 0.01	1.63 ± 0.32	0.79 ± 0.45	c	0.65 ± 0.24	-0.44 ± 0.36	0.80 ± 0.28
SMG 29	-3.3 ± 0.9	a	30.8 ± 1.7	203.8 ± 23.3	a	220.0 ± 5.5	11.3 ± 1.0	a	0.25 ± 0.02	b	0.46 ± 0.03	0.34 ± 0.04	0.20 ± 0.02	2.07 ± 0.49	1.74 ± 0.34	d	0.69 ± 0.09	-0.15 ± 0.24	0.45 ± 0.18
SMG 3	-4.8 ± 0.4	b	31.7 ± 0.6	163.4 ± 18.7	b	240.3 ± 6.6	9.4 ± 1.5	b	0.22 ± 0.03	b	0.49 ± 0.03	0.29 ± 0.03	0.21 ± 0.01	1.37 ± 0.13	0.54 ± 0.56	c	0.68 ± 0.24	-0.49 ± 0.30	0.81 ± 0.15
SMG 30	-4.7 ± 0.3	b	32.9 ± 0.8	252.5 ± 21.9	a	227.3 ± 4.9	11.9 ± 0.9	a	0.27 ± 0.02	a	0.46 ± 0.02	0.33 ± 0.02	0.22 ± 0.01	1.59 ± 0.19	2.05 ± 0.82	d	0.62 ± 0.42	-2.23 ± 1.48	2.61 ± 1.61
SMG 31	-5.3 ± 0.4	b	31.1 ± 0.9	192.6 ± 23.4	a	228.0 ± 4.2	9.6 ± 0.8	b	0.22 ± 0.02	b	0.50 ± 0.01	0.29 ± 0.02	0.21 ± 0.01	1.42 ± 0.16	0.94 ± 0.15	c	2.57 ± 1.74	-5.30 ± 4.36	3.73 ± 2.64
SMG 4	-5.0 ± 0.5	b	29.9 ± 1.7	217.0 ± 28.3	a	224.5 ± 4.2	10.9 ± 1.0	a	0.25 ± 0.02	b	0.47 ± 0.02	0.33 ± 0.02	0.21 ± 0.01	1.72 ± 0.30	0.84 ± 0.46	c	0.34 ± 0.17	0.20 ± 0.48	0.45 ± 0.33
SMG 5	-5.4 ± 0.6	b	30.1 ± 1.2	174.5 ± 19.1	b	234.8 ± 9.0	9.4 ± 1.1	b	0.21 ± 0.02	c	0.50 ± 0.02	0.30 ± 0.03	0.20 ± 0.01	1.55 ± 0.21	0.81 ± 0.32	c	1.69 ± 1.03	-0.40 ± 0.60	-0.29 ± 0.69
SMG 6	-6.0 ± 0.4	b	32.1 ± 0.9	170.3 ± 17.7	b	232.6 ± 7.2	9.0 ± 1.1	b	0.21 ± 0.02	c	0.50 ± 0.02	0.29 ± 0.02	0.21 ± 0.01	1.39 ± 0.13	0.74 ± 0.26	c	0.50 ± 0.25	-0.58 ± 0.47	1.09 ± 0.36
SMG 7	-5.0 ± 0.6	b	31.9 ± 1.5	188.3 ± 16.8	b	223.1 ± 8.3	11.5 ± 1.2	a	0.25 ± 0.02	b	0.47 ± 0.02	0.31 ± 0.02	0.23 ± 0.01	1.44 ± 0.18	1.72 ± 0.26	d	0.31 ± 0.06	-0.18 ± 0.18	0.88 ± 0.15
SMG 8	-5.0 ± 0.3	b	30.5 ± 1.2	194.7 ± 17.8	a	218.7 ± 4.9	10.8 ± 0.8	a	0.24 ± 0.02	b	0.48 ± 0.02	0.31 ± 0.02	0.21 ± 0.01	1.59 ± 0.18	0.73 ± 0.16	c	0.49 ± 0.42	-4.09 ± 2.53	4.60 ± 2.60
SMG 9	-5.4 ± 0.2	b	34.7 ± 2.1	192.0 ± 22.5	a	230.2 ± 5.6	9.9 ± 1.5	b	0.22 ± 0.03	b	0.49 ± 0.02	0.29 ± 0.02	0.22 ± 0.01	1.35 ± 0.14	1.45 ± 0.15	d	0.89 ± 0.23	-0.33 ± 0.19	0.44 ± 0.29
SMR 168	-3.7 ± 0.5	a	34.9 ± 1.8	258.7 ± 20.9	a	222.2 ± 6.0	13.8 ± 1.2	a	0.30 ± 0.02	a	0.44 ± 0.02	0.36 ± 0.04	0.20 ± 0.02	2.13 ± 0.51	1.64 ± 0.28	d	0.60 ± 0.13	-1.11 ± 1.00	1.51 ± 0.89
SMR 169	-4.3 ± 0.4	b	30.8 ± 1.2	162.6 ± 10.7	b	223.1 ± 9.1	9.0 ± 0.7	c	0.20 ± 0.01	c	0.50 ± 0.02	0.29 ± 0.03	0.21 ± 0.01	1.65 ± 0.47	-2.40 ± 0.15	b	0.62 ± 0.14	-1.01 ± 0.86	1.39 ± 0.93
SMR 190	-3.9 ± 0.5	a	32.1 ± 1.8	180.3 ± 16.2	b	229.7 ± 8.7	9.8 ± 0.9	b	0.22 ± 0.02	b	0.50 ± 0.02	0.29 ± 0.03	0.21 ± 0.01	1.50 ± 0.28	3.11 ± 2.01	d	0.68 ± 0.20	-0.16 ± 0.45	0.48 ± 0.32
SMR 191	-3.7 ± 0.7	a	33.0 ± 1.2	185.3 ± 18.1	b	235.3 ± 5.5	8.8 ± 0.7	c	0.21 ± 0.02	c	0.52 ± 0.01	0.25 ± 0.01	0.23 ± 0.01	1.11 ± 0.09	0.60 ± 0.98	c	0.69 ± 0.12	-0.10 ± 0.19	0.40 ± 0.11
