# Supplementary figures and images for: Adaptation of Interspecific Mesoamerican Common Bean Lines to Acid Soils and High Temperature in the Amazon Region of Colombia
Source: Plants (Basel). 2021 Nov 9;10(11):2412. doi: 10.3390/plants10112412 (PMC8623317; doi:10.3390/plants10112412)

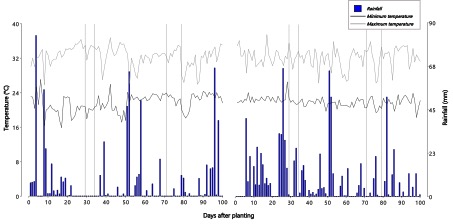

Supplement: Supplementary file 1 [file plants-10-02412-s001.zip › plants-1425338-proofed suppl/Supplementary material 3.jpg]
